# Toxin dimerization and a distinct DNA-binding architecture define chromosomal Phd-Doc regulation

**DOI:** 10.1093/nar/gkag280

**Published:** 2026-03-30

**Authors:** Jin Young Park, Minjeong Kim, Bison Lim, Hyo Jung Kim

**Affiliations:** Medical Affairs, Dong-A ST, 64 Cheonho-daero, Dongdaemun-gu, Seoul 02587, Republic of Korea; College of Pharmacy, Woosuk University, Wanju 55338, Republic of Korea; College of Pharmacy, Woosuk University, Wanju 55338, Republic of Korea; College of Pharmacy, Woosuk University, Wanju 55338, Republic of Korea

## Abstract

Toxin–antitoxin (TA) systems are widespread bacterial regulatory modules, yet the structural diversity of chromosomally encoded TA regulation remains incompletely understood. Here, we show that the *Enterococcus faecalis* Phd-Doc module adopts a regulatory architecture distinct from the canonical *Escherichia coli* paradigm. Structural analyses reveal that toxin neutralization is associated with antitoxin-mediated toxin dimerization rather than direct occlusion of the conserved catalytic motif. In parallel, the N-terminal domain of *E. faecalis* Phd forms a β-sheet-based DNA-binding arrangement that recognizes a single palindromic operator, contrasting with the cooperative dual-operator recognition observed in *E. coli*. Together, these features define an alternative configuration for operator recognition and toxin regulation within a chromosomal TA system. Structure-guided peptide design further demonstrates that the *E. faecalis* Phd-Doc interface can be selectively perturbed, highlighting the potential of exploiting distinct TA architectures for differential functional modulation.

## Graphical Abstract

**Graphical Abstract:**
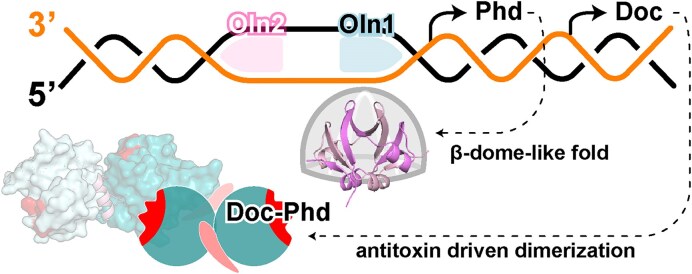

## Introduction

Multidrug-resistant (MDR) *Enterococcus faecalis* has emerged as a formidable pathogen responsible for life-threatening infections, including bacteremia and endocarditis [1, 2]. Its remarkable ability to acquire and disseminate antibiotic resistance determinants has severely undermined the efficacy of conventional therapies, culminating in the emergence of vancomycin-resistant strains that pose a serious public health threat [3]. These challenges highlight the urgent need to better understand the molecular systems that regulate bacterial physiology and gene expression under antimicrobial stress.

In particular, dissecting these regulatory pathways provides a foundation for identifying vulnerabilities that could be exploited to develop novel therapeutic strategies.

Toxin–antitoxin (TA) systems are widespread genetic modules found in bacterial and archaeal genomes. Type II TA systems typically consist of a stable toxin protein and a cognate antitoxin that directly neutralizes toxin activity while often repressing transcription of the operon [4]. Although TA systems have been implicated in stress adaptation and persistence, their precise physiological roles remain debated, and null phenotypes are frequently observed in deletion strains [5, 6]. Recent studies have shifted the focus from stress survival models toward roles in genomic conflicts, plasmid maintenance, and defense against mobile genetic elements [7]. Despite ongoing debate regarding their physiological relevance, TA systems exhibit remarkable structural and mechanistic diversity [8–10]. The Phd-Doc module represents one of the best-characterized Type II systems in *Escherichia coli*, where cooperative DNA binding and toxin neutralization have been extensively studied. However, chromosomal TA systems in other bacterial species remain less structurally defined, and the extent to which regulatory architectures diverge is unclear. Here, we investigate the chromosomal Phd-Doc module from *E. faecalis* and uncover a distinct structural and regulatory strategy that contrasts with the canonical *E. coli* system.

Among the diverse TA families, the Phd-Doc module represents a widely used model for studying Type II TA systems. In *E. coli*, the Doc toxin inhibits translation elongation by phosphorylating EF-Tu, thereby blocking aminoacyl-tRNA entry into the ribosome, while the Phd antitoxin counteracts this effect by directly binding to Doc and repressing transcription of the TA operon [11, 12]. Interestingly, *E. faecalis* encodes a Phd-Doc system on its chromosome rather than a plasmid [13]. The *E. faecalis* homologs share limited sequence identity with their *E. coli* counterparts, suggesting potential divergence in both structure and regulatory logic. Despite the prevalence of Phd-Doc systems among gut bacteria, little is known about how these modules function beyond the plasmid paradigm defined by *E. coli* [14, 15]. These differences suggest that the *E. faecalis* system may employ distinct structural strategies for toxin neutralization and transcriptional control.

In this study, we determine high-resolution structures of the *E. faecalis* Phd-Doc module to elucidate its mechanism of regulation. Our data reveal unexpected features of both toxin neutralization and promoter recognition, highlighting differences from the canonical *E. coli* model [16, 17]. By comparing these evolutionarily related systems, we define how diverse structural architectures can accomplish similar regulatory outcomes. These insights advance our understanding of TA system evolution and provide a structural framework for future development of species-selective inhibitors targeting *E. faecalis*.

## Materials and methods

### Preparation of _EF_Doc and _EF_Phd proteins

The coding regions for Doc (159 amino acids) and Phd (79 amino acids) from *E. faecalis* (_EF_Doc and _EF_Phd hereafter) were amplified by polymerase chain reaction (PCR) using gene-specific primers (listed in Supplementary Table S1). For co-expression, _EF_Doc and _EF_Phd were cloned into a pETDuet-1 vector (Novagen), with _EF_Doc fused to an N-terminal 6×His tag (Invitrogen). Additionally, _EF_Doc alone was cloned into pET28a(+) (Novagen), while _EF_Phd constructs (_EF_Phd^1–79^ and _EF_Phd^1–55^) were cloned into pET21a(+) (Novagen). Mutant constructs were generated by site-directed mutagenesis. His54, Lys56, and Lys57 of _EF_Phd cloned in the pETDuet-1 vector were substituted with alanine. In addition, Lys8, Arg10, and Asn14 of _EF_Phd^1–55^ in the pET21a(+) vector were replaced with alanine. All constructs were verified by Sanger sequencing. Recombinant plasmids were transformed into *E. coli* C41(DE3) competent cells. Single colonies were inoculated into LB medium containing the appropriate antibiotics [ampicillin 50 μg/ml for pETDuet-1 and pET21a(+); kanamycin 30 μg/ml for pET28a(+)] and grown at 37°C with shaking at 180 rpm until the optical density at 600 nm (OD_600_) reached 0.5. Protein expression was induced by adding isopropyl β-d-1-thiogalactopyranoside (IPTG) to a final concentration of 1 mM, followed by incubation for an additional 4 h at 37°C. Cells were harvested by centrifugation at 4500 × *g* for 10 min at 4°C, and the pellets were resuspended in lysis buffer (50 mM Tris–HCl, pH 7.5, 500 mM NaCl) supplemented with 1 mM phenylmethylsulfonyl fluoride (PMSF) as a protease inhibitor. Cells were disrupted by sonication on ice using an ultrasonic processor (Cole-Parmer; 5 s pulse on/5 s off, total 10 min, 40% amplitude). The lysate was clarified by centrifugation at 20 000 × *g* for 1 h at 4°C.

The supernatant was loaded onto a Ni-NTA (Ni²⁺-nitrilotriacetic acid) agarose column (Qiagen, Germany; 3 ml resin per liter of culture) pre-equilibrated with lysis buffer. After washing with 10 column volumes of wash buffer (50 mM Tris–HCl, pH 7.5, 500 mM NaCl, 20 mM imidazole), the bound proteins were eluted using elution buffer containing 50 mM Tris–HCl, pH 7.5, 500 mM NaCl, and ∼200 mM imidazole. Elution fractions were analyzed by sodium dodecyl–sulfate polyacrylamide gel electrophoresis (SDS–PAGE) followed by Coomassie Brilliant Blue staining. For further purification and buffer exchange, proteins were subjected to size-exclusion chromatography using a Superdex 75 10/300 GL column (Cytiva) equilibrated with 50 mM Tris–HCl, pH 7.5, and 150 mM NaCl. Fractions containing the target proteins were pooled and concentrated to 10 mg/ml using ultrafiltration devices with a 3 kDa molecular weight cut-off (Millipore). The final protein purity was estimated to exceed 95% by SDS–PAGE densitometry. For complex formation, purified _EF_Phd^1–55^ protein was incubated with double-stranded DNA (Oln1: 5′-TGTATATACA-3′ and Oln2: 5′-ATCCATGAT-3′, sequences shown correspond to one strand) at a 2:1 molar ratio of protein to DNA for 30 min at 20°C. The resulting mixture was subjected to size-exclusion chromatography for complex isolation, followed by crystallization trials.

### Crystallization, data collection, and structure determination

Crystallization trials were carried out at 20°C using the hanging-drop vapor-diffusion method in 48-well VDX plates (Hampton Research). Initial screening was performed using commercial crystallization kits: Index (Hampton Research), JCSG+, Morpheus, MIDAS (Molecular Dimensions), and Wizard I–IV (Emerald BioSystems). For each trial, 1 μl of protein solution or protein–DNA complex, Oln1, was mixed with 1 μl of precipitant solution and equilibrated against 300 μl of the reservoir solution. Optimized crystallization conditions for each protein or complex are summarized in Supplementary Table S1. Prior to data collection, crystals were cryoprotected by transferring them into mother liquor supplemented with 20% (*v/v*) glycerol for ~30–60 s, then flash-cooled in liquid nitrogen. X-ray diffraction data were collected at 100 K at the Pohang Accelerator Laboratory (PAL) beamlines 5C and 11C using PILATUS 6M and EIGER 9M detectors. Diffraction images were indexed, integrated, and scaled with the *HKL3000* program package [18, 19].

The crystal structures were determined by molecular replacement using Phaser within the CCP4 suite, with AI-generated models based on *E. coli* Doc (PDB ID: 3K33) and MazE antitoxin (PDB ID: 2MRN) as search models [17, 20]. Model building was carried out through iterative cycles in COOT, followed by refinement with Refmac5 and Phenix.refine [21–25]. For cross-validation, 5% of the diffraction data were excluded from refinement and used to calculate R_free_. Solvent molecules were added in the later stages of refinement, based on electron density maps at a contour level of 1.0 σ. Final crystallographic statistics are summarized in Supplementary Table S2. Structural alignments and molecular graphics were prepared using PyMOL (http://www.pymol.org).

### Quantification of _EF_Doc kinase activity

To assess _EF_Doc kinase activity, the coding sequence of *E. faecalis* EF-Tu was cloned into a pET21a(+) expression vector and expressed in *E. coli* BL21(DE3) cells. Cells were grown at 37°C to an OD_600_ of 0.5 and induced with 0.5 mM IPTG at 37°C for 4 h. Harvested cells were lysed by sonication in lysis buffer (50 mM Tris–HCl, pH 7.5, 500 mM NaCl), and the lysate was clarified by centrifugation. EF-Tu was purified using Ni²⁺-affinity chromatography followed by size-exclusion chromatography. Purified protein was concentrated and stored at −80°C until use.

_EF_Doc kinase activity was quantified using the ADP-Glo kinase assay kit (Promega) according to the manufacturer’s instructions [26, 27]. The _EF_Phd-_EF_Doc complex was serially diluted two-fold starting from 50 nM in kinase buffer (50 mM Tris–HCl, pH 8.5, 100 mM NaCl, 10 mM MgCl_2_). For each reaction, 2.5 μl of complex solution was mixed with 2.5 μl of 2× master mix containing EF-Tu (final concentration, 10 μM), 200 μM ATP, 100 mM Tris–HCl (pH 8.5), 200 mM NaCl, and 20 mM MgCl_2_ in a white 384-well plate. Reactions were incubated at room temperature for 30 min. A negative control lacking the complex was included. The _EF_Phd H54K56K57A mutant complex was assayed under identical conditions.

To terminate the reaction and deplete remaining ATP, 5 μl of ADP-Glo Reagent was added and incubated at room temperature for 40 min. Subsequently, 10 μl of Kinase Detection Reagent was added to convert ADP to ATP and generate luminescence, followed by an additional 40 min incubation at room temperature. Luminescence was measured using an Infinite 200 PRO multimode plate reader (Tecan) with an integration time of 1 s, a settle time of 50 milliseconds, and automatic attenuation. All assays were performed in three independent biological replicates.

### Electrophoretic mobility shift assay

Electrophoretic mobility shift assays (EMSA) were performed to characterize the binding of _EF_Phd^1–55^ to its cognate promoter DNA. Putative promoter regions for _EF_Phd^1–55^ were identified using the BPROM tool (Softberry; http://www.softberry.com) and palindromic DNA motifs were searched using the EMBOSS palindrome tool (http://www.bioinformatics.nl). Based on this analysis, two palindromic or inverted repeat sequences (Oln1 and Oln2) were detected. Both oligonucleotides were synthesized and HPLC-purified by Bioneer Corporation Korea and annealed according to the manufacturer protocol. Binding reactions were prepared in a buffer containing 50 mM Tris–HCl, pH 7.5, and 150 mM NaCl, with 10 nM dsDNA and increasing concentrations of _EF_Phd^1–55^ protein (0, 100, 200, and 300 nM). To evaluate specific binding residues and the effect of complex formation, the _EF_Phd^1–55^ K8R10N14A mutant and the intact _EF_Phd-_EF_Doc complex were also assayed under identical conditions. Samples were incubated at 20°C for 30 min prior to electrophoresis. The complexes were resolved on 1.5% (*w/v*) agarose gels prepared in 0.5× TBE buffer (45 mM Tris-borate, pH 8.0, and 1 mM ethylenediaminetetraacetic acid) and run at 50 V for 30–40 min. DNA bands were visualized using SYBR Safe (Thermo Fisher Scientific) and imaged with a Gel Doc Go imaging system (Bio-Rad) using Image Lab software. Experiments were performed in three independent replicates.

### Peptide activity assays

Synthetic peptides corresponding to the N-terminal (HQKKLQQMMEN, N-peptide) and C-terminal (SKQKHNELYKELVT, C-peptide) regions were obtained from Anygen and DandiCure Inc., Korea, at 95.5% purity. Peptide identity and purity were confirmed via HPLC and mass spectrometry. Antibacterial activity of the peptides was evaluated using a resazurin-based viability assay in *E. faecalis* and *E. coli* [28, 29]. Overnight cultures were diluted into fresh medium and grown to an OD_600_ of 0.5. Bacterial suspensions were then adjusted to an OD_600_ of 0.05 and exposed to serially increasing concentrations of peptides (40–5000 μg/ml) in 96-well plates. Following incubation, resazurin solution was added to a final concentration of 0.02% (*w/v*), and plates were further incubated at 37°C for 30 min until color development was observed. Absorbance was measured at 570 nm and 600 nm using a Sunrise microplate reader (Tecan). Although high peptide concentrations were required, potential turbidity effects in the resazurin assay were mitigated by dual-wavelength correction and independently assessed by spot assays. The percentage of resazurin reduction, reflecting bacterial metabolic activity, was calculated using the following equation:

%Reduction = 100 × [(A570 (sample) − A570 (blank)) – CF × (A600 (sample) − A600 (blank))]

where A represents absorbance at the indicated wavelength, and CF (correction factor) was calculated from blank wells (CF = A570(blank)/A600(blank)). All experiments were performed in at least three independent biological replicates.

For spot assays, *E. faecalis* protoplasts and *E. coli* spheroplasts were generated as previously described [30–32]. Cells were cultured in LB broth (10 ml) at 37°C to an OD_600_ of 0.5, harvested by centrifugation, and washed twice with an equal volume of protoplast/spheroplast buffer containing 20 mM Tris–HCl, pH 7.0, 500 mM sucrose, 20 mM MgCl_2_, and 10 μg/ml lysozyme. Peptide solutions were prepared at final concentrations of 1250, 2500, and 5000 μg/ml. Ampicillin was used as a control at concentrations of 1.25, 2.5, and 5 μg/ml. For the spot assay, 5 μl aliquots of peptide or antibiotic solutions were spotted onto LB agar plates supplemented with 500 mM sucrose (to maintain osmotic stability for protoplasts). After drying, plates were incubated overnight at 37°C. Growth inhibition and cell viability were assessed by imaging the plates using a Gel Doc Go system. All assays were performed in three independent biological replicates.

## Result

### Solving expression challenges for toxic _EF_Doc and unstable _EF_Phd

Individual expression attempts of _EF_Doc and _EF_Phd failed to yield detectable protein levels. _EF_Doc toxicity impeded its expression, while _EF_Phd was rapidly degraded despite the presence of protease inhibitors. Given prior evidence that Phd mitigates Doc-induced toxicity, co-expression was pursued using separate plasmids with different antibiotic markers; however, protein levels remained suboptimal. To better replicate the native operon structure, both genes were cloned into a single plasmid. This strategy led to markedly improved co-expression with a 1:1 stoichiometry and good solubility, enabling purification. Nevertheless, _EF_Phd degradation was observed in the purification process.

To enhance stability, a truncated _EF_Phd construct (residues 1–55) lacking the disordered C-terminal region was generated. This variant was robustly expressed and purified without detectable degradation, as confirmed by SDS–PAGE (Supplementary Fig. S1). NMR analysis further suggested residual flexibility and minor C-terminal degradation (Supplementary Fig. S2).

### Structural basis of antitoxin-mediated _EF_Doc dimerization

**Figure 1.**
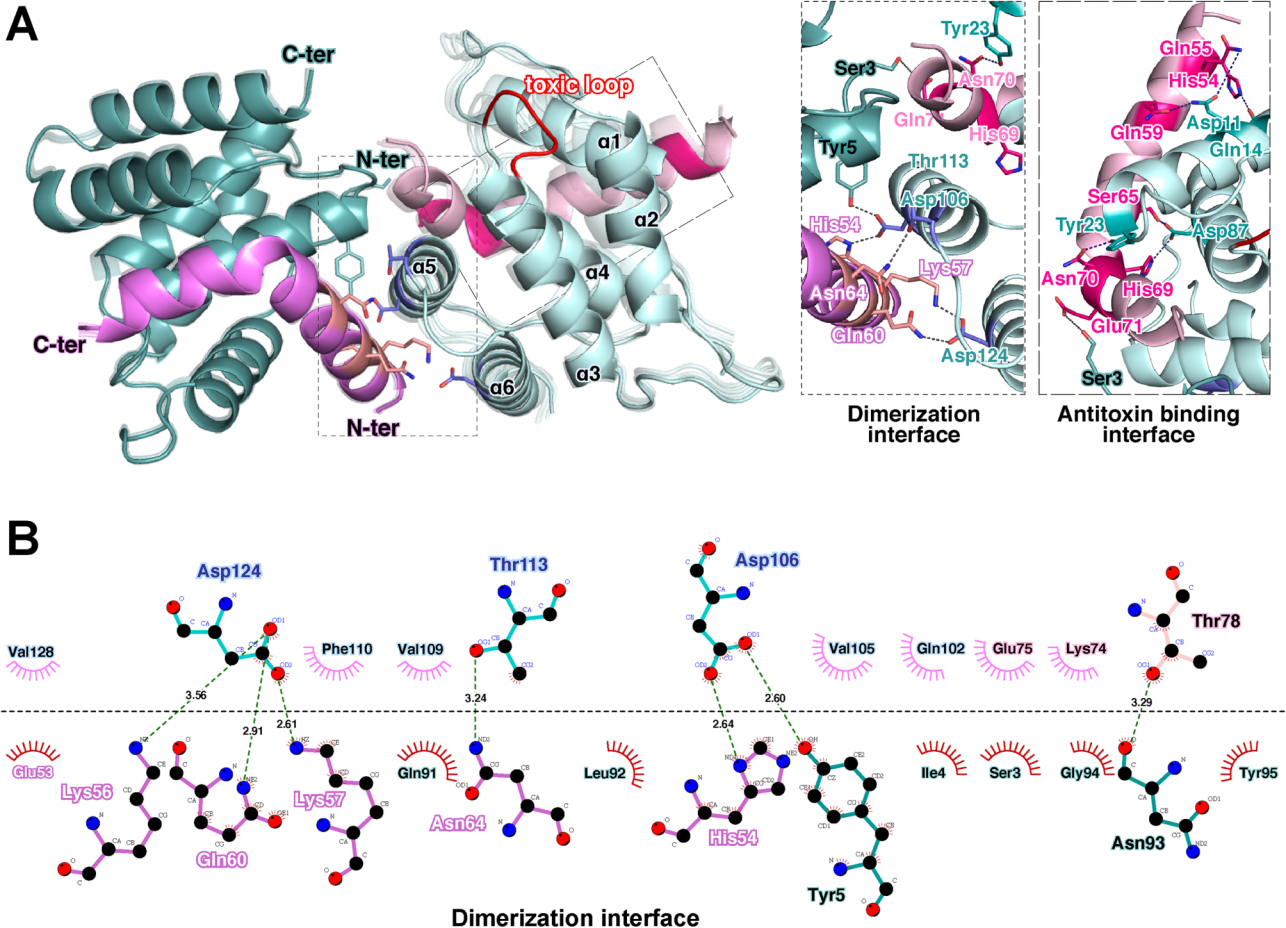
Structural basis of _EF_Phd-_EF_Doc dimerization and antitoxin binding. (**A**) Overall structure of the _EF_Phd-_EF_Doc complex (PDB: 9VZC). _EF_Doc is shown in light teal and cyan (Chain A and C), and _EF_Phd in dark violet and pink (Chain B and D). _EF_Doc forms a dimer, with the C-terminal helix of _EF_Phd (residues 52–79) inserting into each _EF_Doc monomer. Within this segment, residues 54–64 contribute to stabilization of the dimer interface, whereas residues 65–79 (C-peptide) primarily mediate toxin binding. The right panel shows an enlarged view of the dimerization and antitoxin-binding interfaces. Key interface residues are shown as sticks and colored according to chain identity. (**B**) Two-dimensional interaction map of the _EF_Phd-_EF_Doc interface generated using DimPlot. Hydrogen bonds and hydrophobic contacts are indicated. Residues participating in intermolecular interactions are labeled, with color coding corresponding to panel (A).

**Figure 2.**
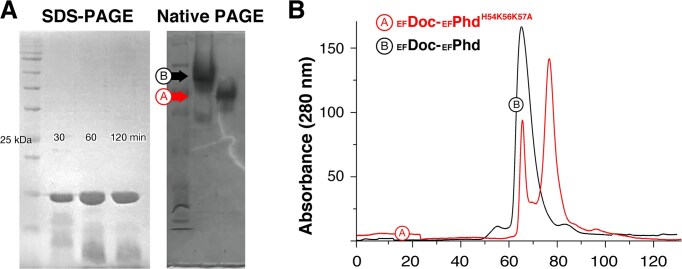
Oligomeric state of the _EF_Phd-_EF_Doc complex. (**A**) SDS–PAGE and native PAGE analysis of the _EF_Phd-_EF_Doc complex. SDS–PAGE was performed at the indicated time points (30, 60, and 120 min) following purification to assess protein stability. Native PAGE shows distinct migration patterns for the wild-type complex (black) and the _EF_Phd interface mutant (H54K56K57A; red), suggesting differences in oligomeric assembly. (**B**) Size-exclusion chromatography profiles of the wild-type _EF_Phd-_EF_Doc complex (black) and the _EF_Phd interface mutant (H54K56K57A; red). The wild-type complex elutes at a volume corresponding to an apparent molecular weight of ∼40 kDa, consistent with a dimeric assembly. In contrast, the mutant displays a major peak shifted toward ∼20 kDa, indicative of a monomeric species and altered oligomeric distribution.

The crystal structure of _EF_Doc was determined at a resolution of 1.8 Å in complex with its cognate antitoxin _EF_Phd. Although the full-length _EF_Phd was used for crystallization, only the C-terminal region (residues 52–79, referred to as _EF_Phd^52–79^) was resolved. _EF_Phd^52–79^ adopts a bent α-helix that inserts into the concave surface of _EF_Doc, resembling the *E. coli* Phd-Doc structures. The overall fold of _EF_Phd-_EF_Doc complex is consistent with that of Fic family proteins. Sequence alignment revealed limited conservation between the *E. faecalis* and *E. coli* Phd-Doc systems. _EF_Doc exhibited low sequence similarity to *E. coli* Doc (∼23% overall similarity), whereas no significant sequence similarity was detected between _EF_Phd and *E. coli* Phd (Supplementary Fig. S3). Despite structural resemblance at the monomer level, the complex interface relies on distinct sequence features (Fig. 1). Structural comparison further revealed differences that may influence complex assembly. In *E. coli*, the Doc α2-helix mediates a relatively flexible interaction with additional *E. coli* Phd, facilitating formation of higher-order multiprotein assemblies. In contrast to the elongated α2-helix of *E. coli* Doc, the corresponding α2-helix of _EF_Doc is shorter and less extended, which may limit accommodation of additional _EF_Phd molecules (Fig. 6). Consistently, no evidence of higher-order complex formation was observed by size-exclusion chromatography or SDS–PAGE (Fig. 2).

In the complex structure, the conserved toxic motif HXFX(D/N)(A/G)NKR remains exposed at the protein surface, and no direct overlap between _EF_Phd and the catalytic region is observed. These observations indicate that steric occlusion of the catalytic motif is unlikely to be the sole mechanism of neutralization. Instead, the complex forms a compact dimeric assembly with a buried surface area of ~730 Å^2^ as estimated by PISA analysis, in which _EF_Phd contributes substantially to stabilization of the dimer interface [33, 34]. Size-exclusion chromatography analysis of the co-expressed complex revealed a single peak corresponding to an apparent molecular weight of ∼40 kDa, consistent with a dimeric assembly. Alanine substitutions at _EF_Phd residues His54, Lys56, and Lys57 altered the oligomeric distribution, resulting in the appearance of a monomeric species (∼20 kDa) in addition to the dimer. Native PAGE analysis corroborated this shift in oligomeric state (Fig. 2).

Comparison of multiple crystal forms indicated that the dimer interface is structurally rigid, whereas peripheral regions exhibit localized flexibility. Structural alignment suggests that residues 54–64 (referred to as the N-peptide) of _EF_Phd contribute to stabilization of the dimer interface, whereas residues 65–79 (referred to as the C-peptide) primarily mediate toxin binding. Together, these results define the structural framework underlying antitoxin-mediated _EF_Doc dimerization.

### Dimerization-dependent functional consequences of _EF_Phd-_EF_Doc complex

**Figure 3.**
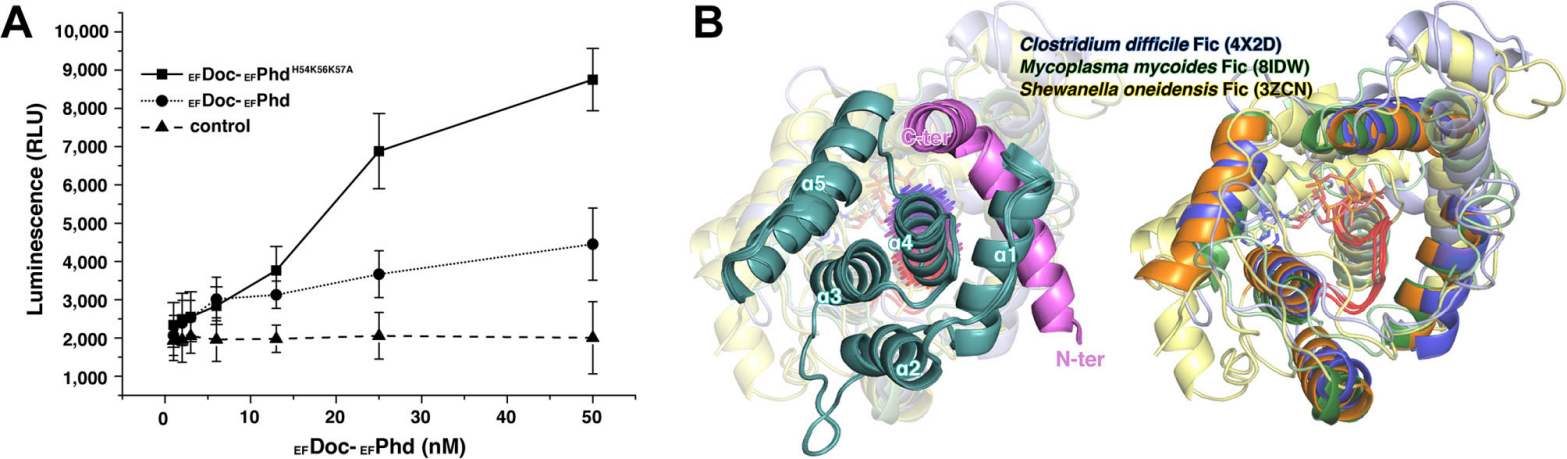
_EF_Doc activity modulated by oligomeric state and structural comparison with Fic family proteins. (**A**) Quantification of _EF_Doc kinase activity using the ADP-Glo kinase assay. Luminescence (relative light units, RLU) is plotted against _EF_Phd-_EF_Doc complex concentration. The _EF_Phd interface mutant complex (H54K56K57A; solid squares) shows increased luminescence relative to the wild-type complex (circles), whereas the negative control lacking the complex (triangles) remains at background levels. Data represent mean ± standard deviation from three independent experiments. (**B**) Structural comparison of _EF_Doc with representative Fic family proteins. (*Left*) _EF_Doc (teal) in complex with _EF_Phd (magenta), with secondary structural elements labeled. The dipole moment of the α4 helix is shown with a background color gradient, with the negatively charged end in red and the positively charged end in blue. (*Right*) superposition of _EF_Doc with Fic proteins from *Clostridium difficile* (PDB: 4 × 2D), *Mycoplasma mycoides* (PDB: 8IDW), and *Shewanella oneidensis* (PDB: 3ZCN), illustrating conservation of the overall Fic fold and catalytic core architecture. In the aligned crystal structures, a γ-phosphate was observed bound to the α4 helix. Helices contributing to the catalytic core, including the α4 helix, are highlighted in darker tones to emphasize structural conservation across Fic family proteins.

We next examined whether _EF_Doc enzymatic activity depends on its oligomeric state. ADP-Glo kinase assays were performed using (i) the wild-type dimeric complex, (ii) a complex containing _EF_Phd interface mutations that partially shift the equilibrium toward a monomeric _EF_Doc population, and (iii) a negative control lacking the complex. The mutant complex exhibited approximately two-fold higher relative luminescence signals, reflecting increased ATP turnover compared to the wild-type dimeric form (Fig. 3A). Because the catalytic residues of _EF_Doc were unaltered in the interface mutant, this result suggests that _EF_Doc activity is modulated by its oligomeric state rather than by disruption of the active site itself.

_EF_Doc belongs to the Fic family of toxins and inhibits translation by transferring a phosphate group from ATP to EF-Tu. Structurally, the γ-phosphate of ATP is positioned near the α4 helix of _EF_Doc, where stabilization of the negative charge is supported by the helix dipole. Proper positioning of the α4 helix is therefore a key determinant of catalytic function. In the complex structure, _EF_Phd contacts regions proximal to the α4 helix, a structural element important for catalytic positioning. Notably, the active site remains solvent-exposed in the complex, and structural superposition with other Fic family proteins indicates that ATP can still be accommodated without major steric clash (Fig. 3B). These observations are not readily explained by a neutralization mechanism based solely on direct active-site occlusion. Instead, the dimeric arrangement of _EF_Doc may influence the spatial accessibility of the toxin and thereby reduce productive interaction with EF-Tu. Together, these findings support a model in which _EF_Doc neutralization may involve antitoxin-driven toxin dimerization and higher-order structural rearrangement rather than direct catalytic blockage. Collectively, our data suggest that _EF_Doc activity is influenced not only by folding and catalytic-site integrity but also by antitoxin-induced quaternary structural organization.

### A distinct β-dome arrangement of _EF_Phd for DNA binding

**Figure 4.**
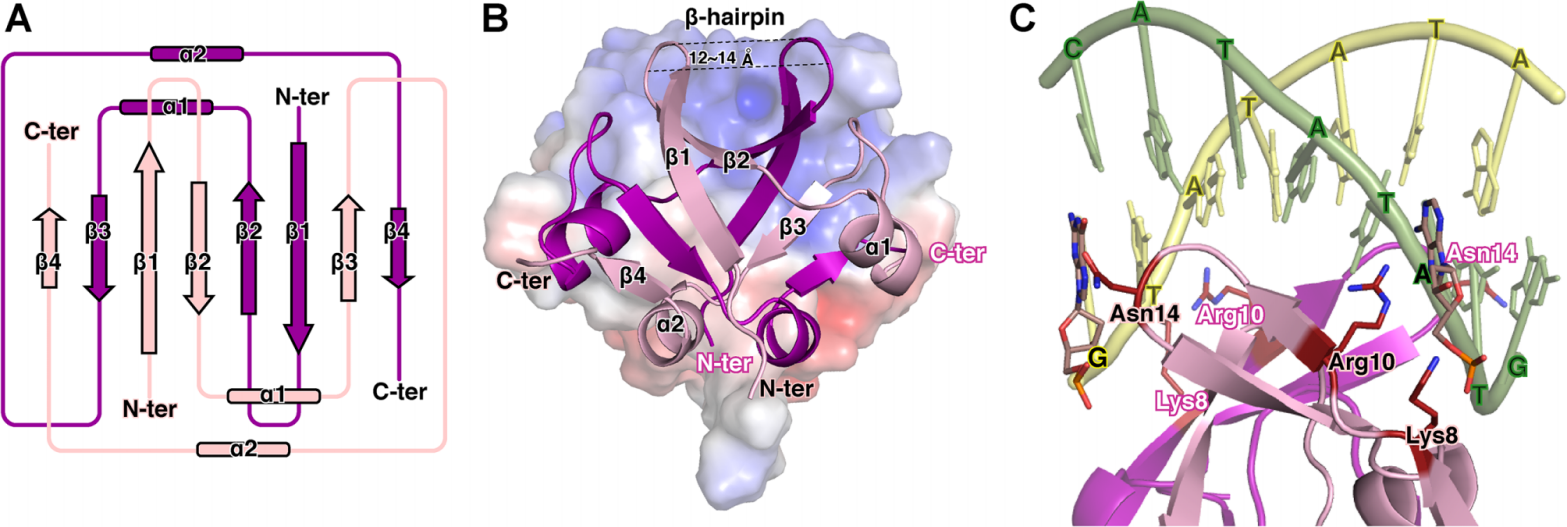
Structural insights into DNA recognition by the β-hairpin motif of _EF_Phd^1–55^. (**A**) Topology diagram of _EF_Phd^1–55^, comprising four β-strands (β1–β4) and two α-helices (α1–α2) arranged in the order β1–β2–α1–β3–α2–β4. (**B**) Ribbon and electrostatic surface representation of the _EF_Phd^1–55^ dimer, illustrating the β-dome-like architecture formed by the antiparallel β-sheet and intercalated helices. The β-hairpin motif (β1–β2) is separated by ∼12–14 Å, consistent with the approximate width of the DNA major groove. Electrostatic potential surface contoured at +10 kT/e (blue) and −10 kT/e (red). (**C**) Structure of the _EF_Phd^1–55^-DNA complex. Putative DNA-interacting residues are shown in stick representation. Lys8 is positioned near the DNA phosphate backbone, whereas Arg10 and Asn14 are located in proximity to nucleobases, suggesting involvement in DNA contact.

The crystal structure of the N-terminal region of _EF_Phd^1–55^ was determined at 2.1 Å resolution and revealed a fold distinct from *E. coli* Phd (Fig. 4). The overall topology is defined by a ββαβαβ arrangement, in which an antiparallel, domed β-sheet associates with that of the adjacent monomer, demonstrating that the protein exists predominantly as a dimer. A similar continuous β-sheet organization across monomers is a common feature of AbrB family proteins, which employ a β-hairpin motif for DNA binding. In these proteins, successive β-strands wrap to form a β-barrel-like structure. In contrast, _EF_Phd^1–55^ possesses an interrupted β-barrel, where α-helices intercalate into the barrel, resulting in a dome-like β-sheet architecture characterized by a convex β-sheet surface reinforced by intercalated helices. The α1 and α2 helices are regularly aligned, and their side chains stabilize the dimeric interface. This arrangement exhibits a modified β-sheet organization compared with other AbrB-family antitoxins such as MazE and PemI [35–38]. Structural similarity searches using DALI identified only weak matches to AbrB-family proteins (*Z*-score ∼3.8), consistent with _EF_Phd adopting a divergent β-sheet-based architecture rather than a canonical AbrB fold. Together with the neighboring dimer, the α1–α2–α2–α1 helical assembly generates a highly negatively charged surface, in stark contrast to the positively charged β-hairpin motif. This bipartite electrostatic organization suggests that _EF_Phd^1–55^ may facilitate DNA binding, with the positively charged face positioned for DNA interaction, while the negatively charged face contributes to spatial orientation of the protein surface (Fig. 4B). We also determined other crystal forms in a different space group, but all structures exhibited the same dimerization mode, with an rmsd of 1.1 Å. Even when one monomer was fixed during alignment, the other remained virtually unchanged, highlighting the rigidity of the dimer and the robustness of its interface.

In the dimeric state, the β-hairpins are separated by ∼12–14 Å, a distance closely matching the width of the DNA major groove (∼12 Å). This geometry allows the dimer to recognize a DNA-binding site spanning ∼8 base pairs. We identified a 10-bp palindromic sequence near the TATA box and crystallized _EF_Phd^1–55^ in complex with double-stranded DNA. Although the structure was determined at limited resolution, key residues were observed near the DNA in positions consistent with nucleotide recognition. Lys8 on the β1-strand is positioned near the DNA backbone, whereas Arg10 and Asn14 are located in proximity to nucleobases, suggesting potential involvement in base-contact interactions. While numerous positively charged amino acids are enriched at the DNA binding region, only a few play a key role in base recognition (Fig. 4C). These findings suggest that the _EF_Phd^1–55^ β-hairpin mediates DNA binding primarily through electrostatic interactions. This contrasts with *E. coli* Phd, which adopts a βααββ DNA-binding fold distinct from the _EF_Phd architecture.

### _EF_Phd selectively recognizes a single palindromic operator

**Figure 5.**
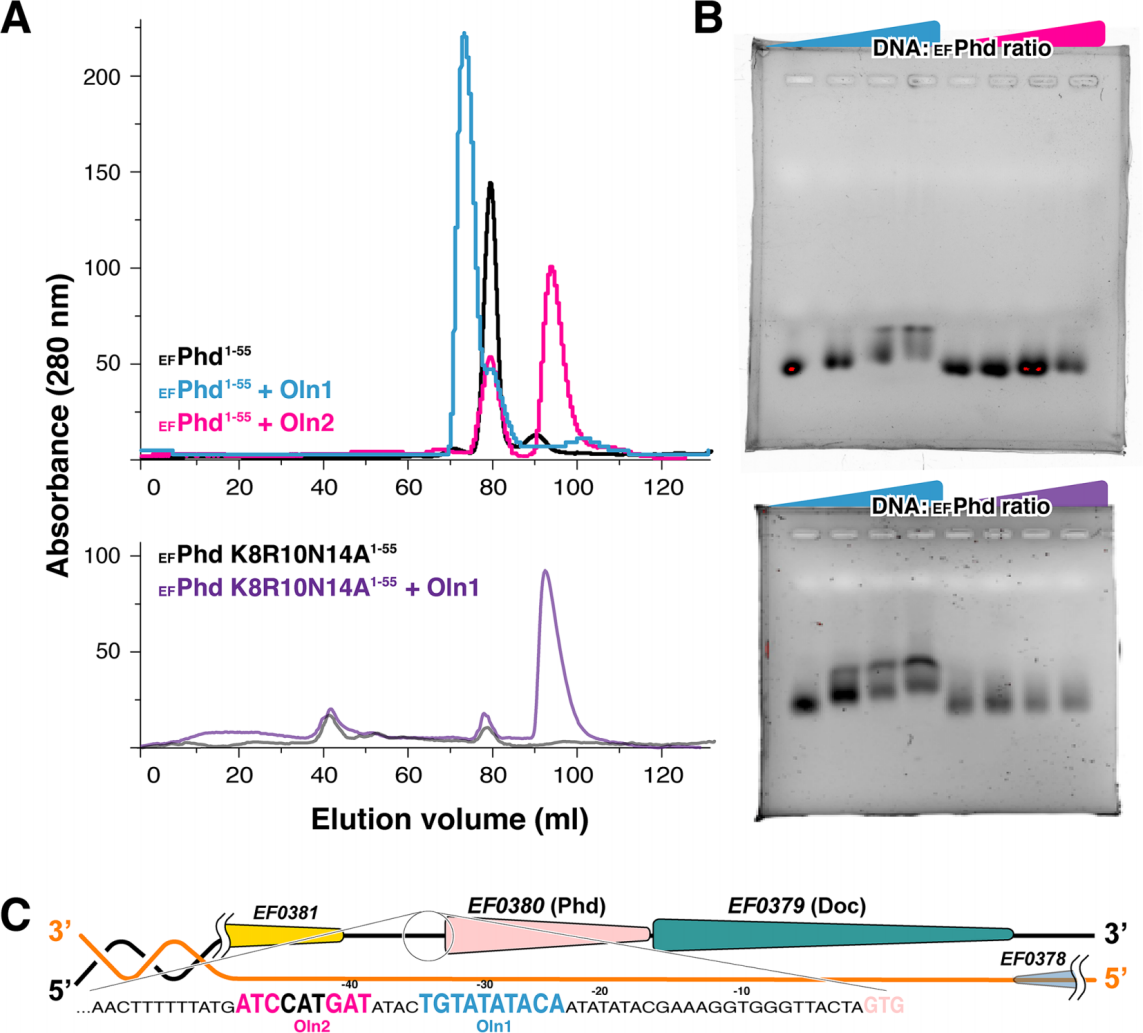
Specific palindromic DNA recognition by _EF_Phd^1–55^. (**A**) Size-exclusion chromatography analysis of _EF_Phd^1–55^ in the absence or presence of DNA oligonucleotides. Elution profiles are shown for protein alone (black), protein with Oln1 (sky blue), and protein with Oln2 (magenta). Addition of Oln1 results in a shift of the _EF_Phd^1–55^ peak toward a lower elution volume, indicative of higher-molecular-weight complex formation, with a minor peak corresponding to excess unbound DNA. In contrast, Oln2 does not induce a detectable shift, and the profile remains similar to that of the free protein. The _EF_Phd^1–55^ (K8R10N14A) mutant does not exhibit a detectable shift in the presence of Oln1 (Supplementary Fig. S4). (**B**) EMSA of _EF_Phd^1–55^ with Oln1 and Oln2. A clear concentration-dependent mobility shift is observed with Oln1, whereas Oln2 shows no detectable shift under the tested conditions. _EF_Phd^1–55^ (K8R10N14A) mutant fails to produce a band shift with Oln1, consistent with the size-exclusion chromatography analysis in panel (A). (**C**) Schematic representation of the _EF_Phd-_EF_Doc operon, indicating the positions of the identified palindromic elements Oln1 and Oln2 relative to the promoter region.

In Type II TA systems, the antitoxin typically binds specific DNA sequences to repress transcription of the toxin gene. Although _EF_Phd is classified as a Phd-type antitoxin, its architecture differs from the canonical *E. coli* Phd fold and is more closely aligned with AbrB/MazE/MraZ-like DNA-binding domains [36, 39, 40]. Proteins in this structural class commonly function as dimers and recognize palindromic DNA sequences. Given that _EF_Phd also forms a dimer, we examined whether _EF_Phd recognizes palindromic operator sequences. Analysis of the *E. faecalis* promoter region identified two palindromic elements near the TATA box: a perfect 10-bp palindromic sequence (Oln1) and a 3-bp inverted repeat separated by 3-bp spacer (Oln2) (Fig. 5C). Because the C-terminal region of _EF_Phd is not involved in DNA binding, we used the isolated N-terminal DNA-binding domain (_EF_Phd^1–55^) to characterize DNA interaction.

**Figure 6.**
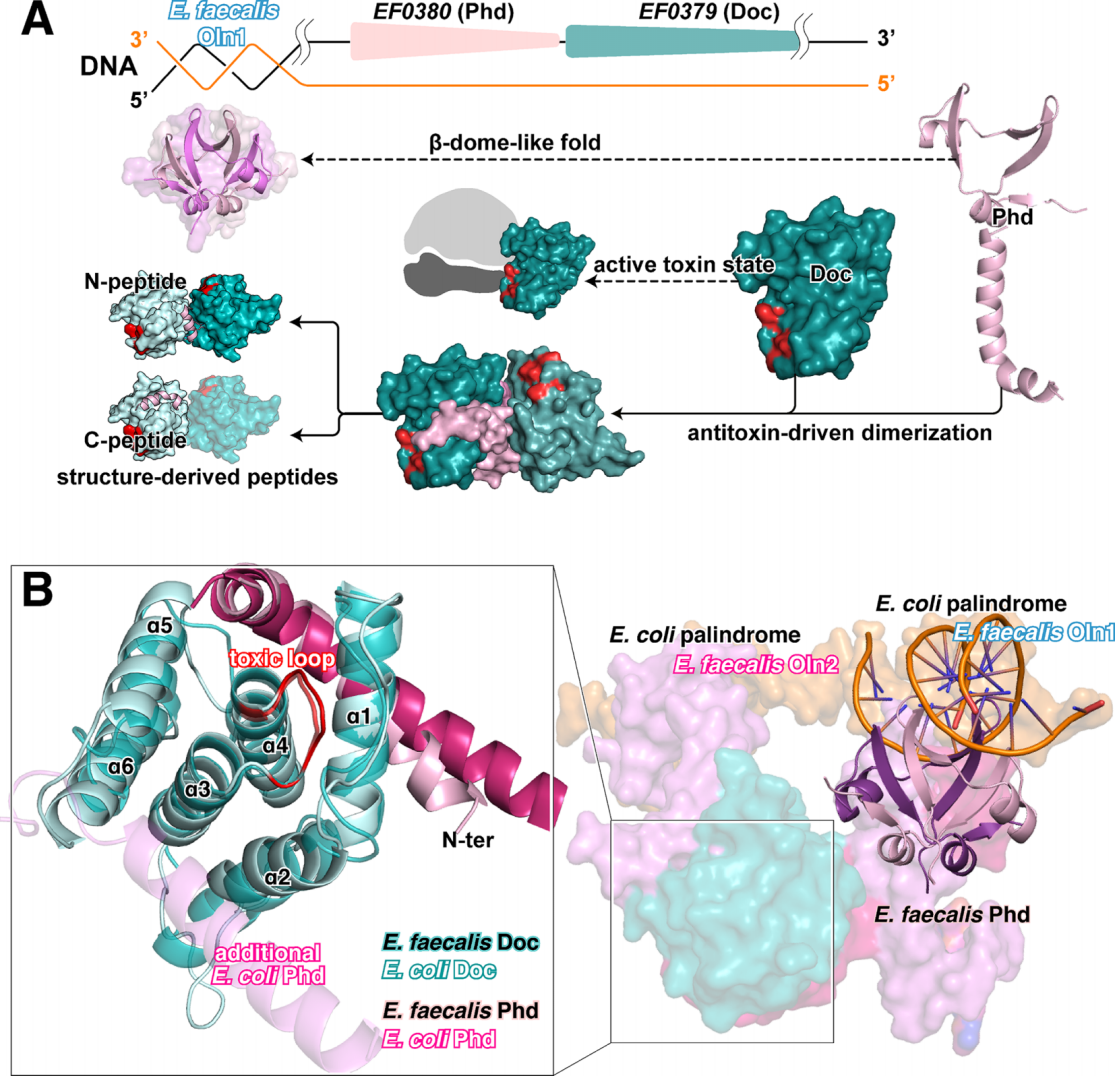
Structural and regulatory distinctions between the *E. faecalis* and *E. coli* Phd-Doc systems. (**A**) Schematic representation of the regulatory architecture of the _EF_Phd-_EF_Doc module. The N-terminal domain of _EF_Phd adopts a β-dome-like fold and associates with the promoter region containing the Oln1 palindromic sequence. _EF_Doc is depicted in a monomeric state associated with higher catalytic activity, whereas binding of _EF_Phd stabilizes a dimeric assembly correlated with reduced activity. The structure-guided design of N-terminal and C-terminal peptides derived from _EF_Phd is also illustrated. (**B**) Structural comparison with the canonical *E. coli* Phd-Doc system. In *E. coli*, the Phd-Doc complex engages two adjacent palindromic DNA sites (shown as light-colored surface representations). In contrast, the *E. faecalis* system selectively recognizes a single palindromic operator and remains predominantly dimeric assembly. The structural basis underlying this distinction, including differences in the α2 helix region of Doc, is highlighted in the enlarged inset.

Size-exclusion chromatography and EMSA demonstrated that _EF_Phd^1–55^ binds specifically to the Oln1, whereas no detectable interaction was observed with the Oln2. This selectivity was maintained across DNA concentrations, with binding observed only for Oln1. To validate the structural basis of DNA recognition, residues Lys8, Arg10, and Asn14—positioned near the DNA interface in the crystal structure—were substituted with alanine (K8R10N14A). This triple mutant failed to bind DNA in EMSA analysis, indicating that these residues contribute substantially to DNA recognition (Fig. 5A and B).

In the *E. coli* Phd-Doc system, Doc provides two antitoxin-binding interfaces, enabling Phd dimers to cooperatively engage two adjacent palindromic operators. In contrast, _EF_Doc forms a toxin dimer that does not support cooperative dual-operator binding. Consistent with this structural difference, EMSA showed that the intact _EF_Phd-_EF_Doc complex does not bind DNA, whereas the isolated N-terminal domain of _EF_Phd^1–55^ readily binds the operator (Supplementary Fig. S4). These findings indicate that _EF_Phd recognizes a single palindromic operator and does not exhibit detectable DNA binding when complexed with _EF_Doc under our experimental conditions.

### Rational design of _EF_Phd-derived peptides reveals selective antibacterial effects in *E. faecalis*

The well-characterized Phd-Doc module contributes to the regulation of toxin activity and expression. This system operates through two coordinated mechanisms: direct toxin neutralization via tight TA binding and transcriptional repression mediated by sequence-specific DNA recognition [41, 42]. Structural analysis revealed that the _EF_Phd-_EF_Doc complex displays distinct architectural features compared to canonical Phd-Doc systems. Notably, _EF_Phd adopts a β-dome-like fold and utilizes a limited number of base-specific contacts for DNA recognition, suggesting relatively low sequence specificity at the DNA-binding interface. These features suggest that targeting the DNA-binding interface may complicate the achievement of species selectivity. In contrast, the _EF_Phd-_EF_Doc interface is defined by extensive and highly specific interactions. Based on this structural distinction, we prioritized a strategy aimed at perturbing toxin neutralization by targeting the _EF_Phd-_EF_Doc interaction surface rather than the DNA-binding interface.

**Figure 7.**
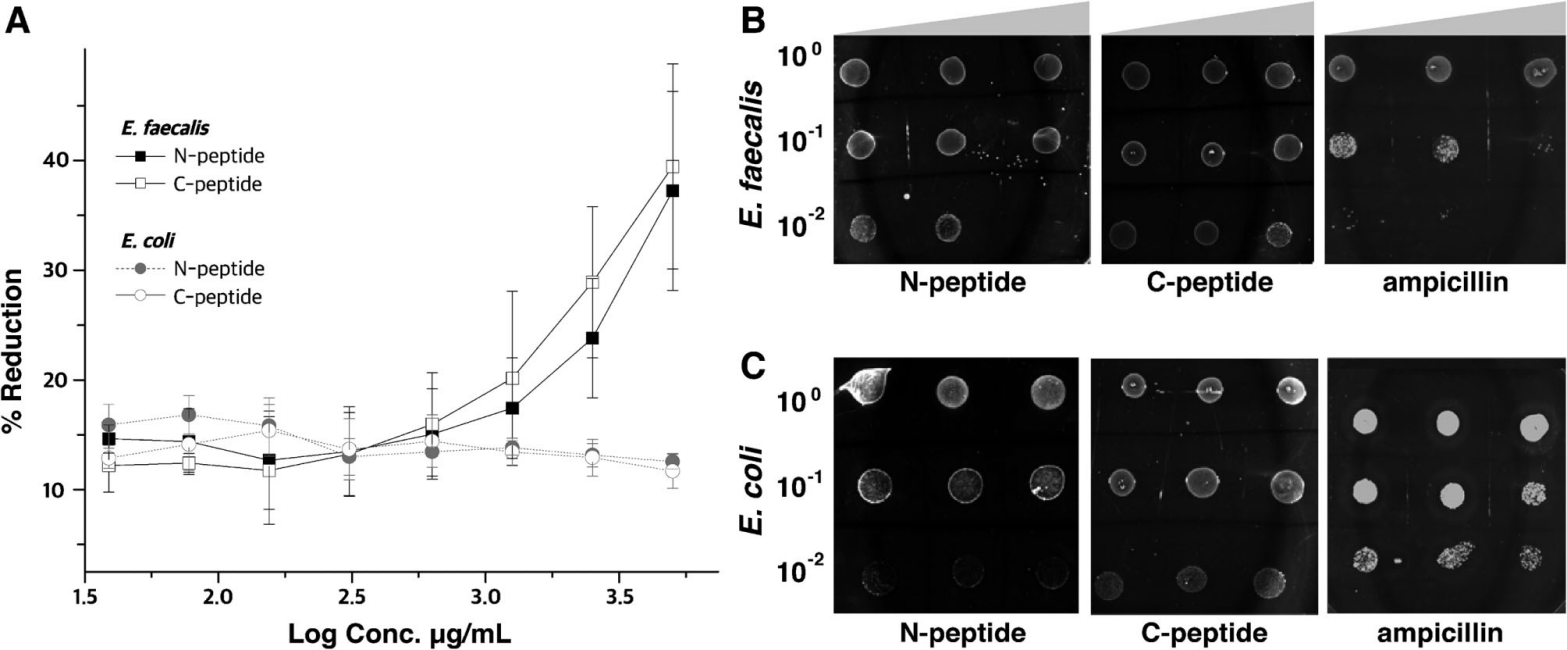
Species-specific growth inhibition of *E. faecalis* by _EF_Phd-derived peptides. (**A**) Resazurin-based cell viability assay showing selective, concentration-dependent inhibition of bacterial growth by N- and C-peptides. Percent resazurin reduction (%) is plotted against log peptide concentration (μg/ml). Both peptides exhibited increased inhibitory effects in *E. faecalis*, whereas no substantial inhibition was observed in *E. coli* under the same conditions. Data represent mean ± standard deviation from three independent biological experiments. (**B**) Spot assay assessing peptide-mediated growth inhibition against *E. faecalis* protoplasts. Serial 10-fold dilutions (10^0^∼10^−2^) were spotted onto agar plates in the presence of N-peptide, C-peptide, or ampicillin as a positive control. Both peptides reduced colony formation in a concentration-dependent manner, whereas ampicillin inhibited growth at lower concentrations. Peptides were tested at 1250, 2500, and 5000 μg/ml and ampicillin was tested at 1.25, 2.5, and 5 μg/ml. (**C**) Spot assay against *E. coli* spheroplasts under identical conditions. Ampicillin produced clear growth inhibition, whereas neither N-peptide nor C-peptide resulted in detectable suppression of colony formation across the tested concentrations. Peptides and ampicillin were tested at the same concentrations as in panel (**B**).

Guided by the bound _EF_Phd structure, two structure-derived peptides were designed and synthesized. The N-terminal peptide (N-peptide) was derived from the region involved in _EF_Doc dimerization, whereas the C-terminal peptide (C-peptide) was derived from the _EF_Phd-_EF_Doc binding surface. To evaluate antibacterial activity, dose-dependent growth inhibition assays were performed using *E. faecalis* and *E. coli* under whole-cell conditions. Both peptides exhibited a concentration-dependent inhibition trend in *E. faecalis*; however, complete growth suppression was not observed, and minimum inhibitory concentrations (MICs) could not be determined within the tested range (Fig. 7A). Under identical conditions, no significant inhibitory effect was observed in *E. coli*. To assess whether cell wall permeability influences peptide activity, cell wall-deficient forms (protoplasts/spheroplasts) of *E. faecalis* and *E. coli* were generated and subjected to spot assays. Under these conditions, both peptides produced clear growth inhibition in *E. faecalis* protoplasts, whereas no inhibitory effect was detected in *E. coli* (Fig. 7B and C). The observed inhibition occurred at relatively high concentrations compared to conventional antibiotics, indicating limited potency. The selective growth inhibition observed in *E. faecalis* is compatible with the structural origin of the peptides, although additional factors such as cellular permeability may also contribute.

## Discussion

TA systems are widespread genetic modules found in bacterial genomes and have been implicated in diverse cellular processes. Although their precise physiological roles remain debated, TA modules share a conserved architectural logic in which a toxin is neutralized by a cognate antitoxin that can also repress operon transcription. The Phd-Doc module, initially characterized in the P1 phage of *E. coli*, has since been identified in diverse bacterial lineages, including chromosomally encoded systems in clinically relevant pathogens such as *Salmonella* and *Enterococcus* [43, 44]. Understanding how these homologous modules are structurally organized across species is therefore essential for clarifying how conserved TA logic can be adapted to different genomic contexts.

Despite being classified within the same Phd-Doc family, the *E. faecalis* and *E. coli* systems differ markedly in primary sequence, tertiary fold, quaternary assembly, and regulatory logic. Such divergence illustrates how homologous TA modules can be evolutionarily rewired while preserving a common functional theme. Notably, _EF_Doc retains the core features of the Fic toxin family, whereas _EF_Phd adopts a DNA-binding architecture more reminiscent of AbrB-like regulators, suggesting that TA modules may incorporate structurally distinct regulatory elements to generate new control strategies. This modular flexibility highlights the remarkable evolutionary plasticity of chromosomal TA systems and underscores the structural diversity that can arise within a seemingly conserved TA framework.

Rather than representing a uniform regulatory framework, homologous Phd-Doc modules appear capable of substantial structural diversification while preserving the core TA logic. This evolutionary flexibility likely reflects adaptation to chromosomal context and species-specific regulatory demands. Although the peptide-derived inhibitors described here exhibit limited potency, their selective activity toward *E. faecalis* highlights the potential of exploiting lineage-specific structural interfaces for structure-guided targeting strategies. Further optimization of interface-targeted strategies would provide a structure-guided framework for exploring structurally divergent TA interfaces.

## Supplementary Material

gkag280_Supplemental_File

## Data Availability

The structural data supporting this study are available in the Protein Data Bank (PDB) under accession numbers https://doi.org/10.2210/pdb9vzc/pdb, https://doi.org/10.2210/pdb9vyb/pdb, and https://doi.org/10.2210/pdb9vx7/pdb.
