# Supplementary material for: Toxin dimerization and a distinct DNA-binding architecture define chromosomal Phd-Doc regulation
Source: Nucleic Acids Res. 2026 Mar 30;54(6):gkag280. doi: 10.1093/nar/gkag280 (PMC13034048; doi:10.1093/nar/gkag280)
Supplement: gkag280_Supplemental_File [file gkag280_supplemental_file.pdf]

Supplementary Figure 1.

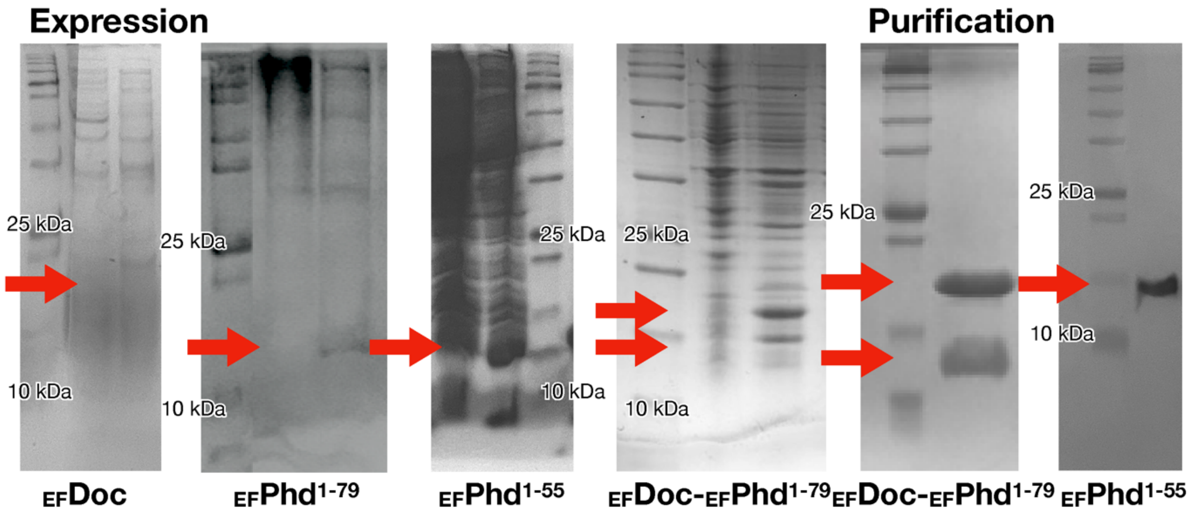

Supplementary Figure 1. Expression and purification of $_{EF}$Doc and $_{EF}$Phd

(Left) SDS-PAGE analysis of protein expression. Expression levels of individually expressed $_{EF}$Doc, $_{EF}$Phd$^{1-79}$, $_{EF}$Phd$^{1-55}$, and co-expressed $_{EF}$Doc-$_{EF}$Phd$^{1-79}$ are shown. Red arrows indicate the expected molecular weights of the respective proteins. While $_{EF}$Doc alone showed low expression level, whereas $_{EF}$Phd$^{1-79}$, $_{EF}$Phd$^{1-55}$, and $_{EF}$Doc-$_{EF}$Phd$^{1-79}$ were expressed to detectable levels. However, $_{EF}$Phd$^{1-79}$ was prone to degradation and not suitable for further purification. *(Right)* SDS-PAGE analysis showing the purified $_{EF}$Doc-$_{EF}$Phd$^{1-79}$ fusion protein and $_{EF}$Phd$^{1-55}$.

Supplementary Figure 2.

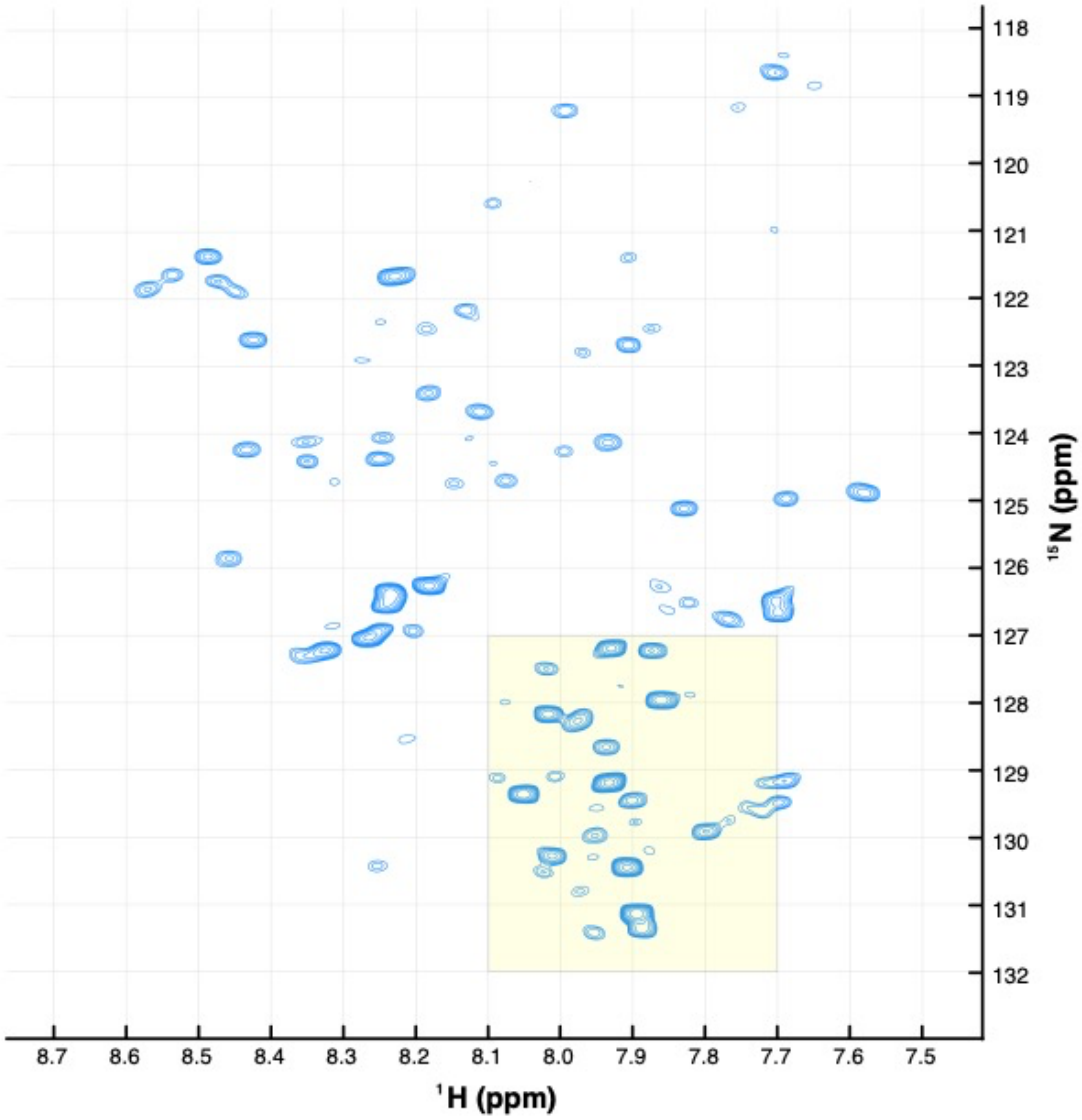

Supplementary Figure 2. 2D [^{1}H-^{15}N] HSQC spectrum of $_{EF}$Phd$^{1\text{-}55}$

The expected number of backbone amide resonances for the $_{EF}$Phd$^{1\text{-}55}$ construct is approximately 55. However, a greater number of peaks were observed, particularly clustered in the highlighted region where resonances from flexible C-terminal residues are typically detected. This pattern is consistent with partial degradation and the presence of truncated species in the sample, potentially arising from C-terminal cleavage.

Supplementary Figure 3.

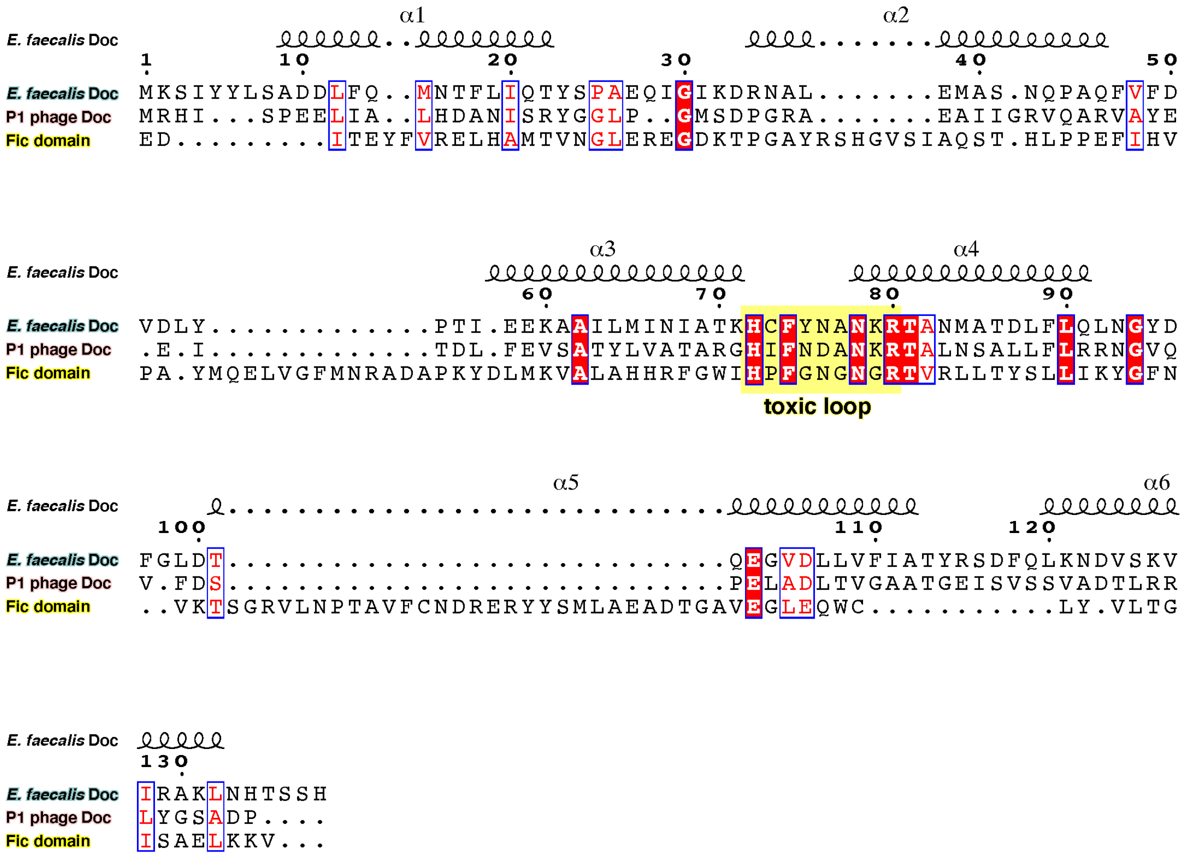

Supplementary Figure 3. Sequence comparison of $_{EF}$Doc, *E. coli* Doc, and the Fic domain consensus

Multiple sequence alignment of $_{EF}$Doc, *E. coli* Doc, and the Fic domain consensus sequence is shown. Secondary structure elements of $_{EF}$Doc are indicated above the alignment. $_{EF}$Doc and *E. coli* Doc share ~23% overall similarity, with conservation largely restricted to the catalytic motif (highlighted in yellow). Conserved residues across the aligned sequences are boxed in red.

Supplementary Figure 4.

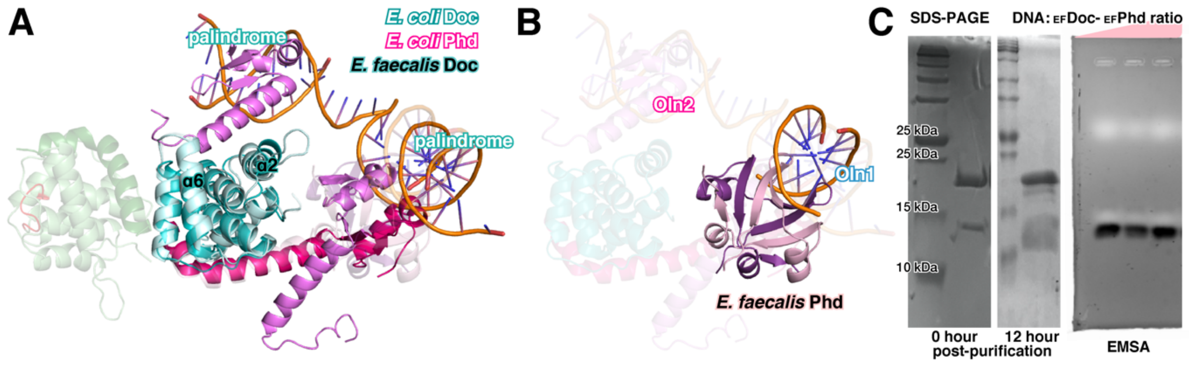

Supplementary Figure 4. Structural comparison of ${}_{EF}$Phd-${}_{EF}$Doc with *E. coli* Phd-Doc assembly and DNA interaction analysis

(A) Structure of the *E. coli* Phd2-Doc (P2D) complex, in which two Phd molecules are anchored by a single Doc and recognize two distinct sites on continuous DNA. The extended α6-helix is predicted to sterically clash with the additional *E. coli* Phd, and the supporting α2-helix is shorter and less ordered in ${}_{EF}$Doc. Thus, ${}_{EF}$Doc is structurally unable to accommodate the additional helix. The α2 and α6 helices are labeled. The ${}_{EF}$Phd-${}_{EF}$Doc complex was overlaid in the background with a light color.

(B) ${}_{EF}$Phd recognizes a single operator region and exhibits a DNA-binding motif distinct from that of *E. coli*. Structural comparison further suggests that the ${}_{EF}$Phd-${}_{EF}$Doc complex does not support formation of higher-order multi-protein assemblies. For reference, the previously reported *E. coli* P2D complex is shown in the background in a faded representation. The *E. coli* Phd structure (PDB ID: 3K33) and its DNA-bound complex (PDB ID: 4ZM0) were used for structural comparison.

(C) EMSA was performed using the ${}_{EF}$Phd-${}_{EF}$Doc complex immediately after purification, under conditions where ${}_{EF}$Phd remained intact. Despite the absence of detectable degradation, no DNA band shift was observed with Oln1.

Supplementary Table 1.

**Oligonucleotide primers**	
**Doc in pET28a**	ggaattc**catatg**aaaagtatttattatttatcagcgg
	ccgccg**ctcgag**ttagtgagaagaagtatg
**Phd in pET21a**	ggaattc**catatg**gtggaaatcaaagaaag
	ccgccg**ctcgag**ttatttagtaaccaattc
**Phd$^{1-55}$ in pET21a**	gaa**catatg**gaaatcaaagaaagaaaac
	gg**ctcgag**gtaaccaattctttgtac
**Doc+Phd in pETDuet-1**	gcggcc**ccatgg**aaaagtatttattatttatc
	ccgccg**ctcgag**ttagtgagaagaagtatg
	ggaattc**catatg**gtggaaatcaaagaaag
	ccgccg**ctcgag**ttatttagtaaccaattc
**N14A Phd$^{1-55}$ in pET21a**	cttagaaaagttgggg**gct**tctgttgttatgac
	gtcataacaacaga**agc**cccaacttttctaag
**K8R10A Phd$^{1-55}$ in pET21a**	gaaatcaaagaaaga**gca**ctt**gca**aaagttggggcttctg
	aagccccaactttt**tgc**aag**tgc**tctttctttgatttccac
**H54K56K57 in Phd+Doc in pETDuet-1**	atatgtcagaa**gct**caa**gcagca**ttacaacaaatgatggagaactca
	atttgttgtaa**tgctgc**ttg**agc**ttctgacatatttttttaacaataatatc
**Crystallization conditions**	
**$_{EF}$Phd-$_{EF}$Doc**	0.05 M citric acid, 0.05 M Bis-Tris propane, pH 5.0, 16% (*w/v*), PEG 3350
**$_{EF}$Phd$^{1-55}$**	0.12 M ethylene glycol, 0.1 M Tris-HCl, pH 8.5, 6% (*w/v*) PEG 8000, 12% (*v/v*) ethylene glycol
**$_{EF}$Phd$^{1-55}$ with DNA**	0.2 M ammonium sulfate, 0.1 M Bis-Tris, pH 5.5, 25% (*w/v*) PEG 3350

Supplementary Table 1. Oligonucleotide primers and crystallization conditions employed in this study

Oligonucleotide primers used for cloning and site-directed mutagenesis are listed. Restriction enzyme recognition sites are indicated in bold. For mutagenesis primers, the substituted

nucleotides corresponding to the intended amino acid changes are shown in bold. For each construct, the forward primer is presented in the upper row and the reverse primer in the lower row. Crystallization conditions for each crystal form are also included.

Supplementary Table 2.

	$_{EF}$Phd$^{52\text{-}79}$-$_{EF}$Doc (9VZC)	$_{EF}$Phd$^{1\text{-}55}$ (9VYB)	$_{EF}$Phd$^{1\text{-}55}$ with DNA (9VX7)
**Data collection**			
Beamline	PAL-11C	PAL-5C	PAL-11A
Wavelength (Å)	0.98	0.98	0.99
Resolution range (Å) [a]	58.3-1.8	27.0-2.1	48.5-4.85
Space Group	$P2_1$	$P2_12_12_1$	$P6_4$
Unit cell parameters (Å)	a=38.06	a=44.37	a=122.52
	b=77.29	b=47.76	b=122.52
	c=58.30	c=48.35	c=118.92
Observations (total/unique)	48,025 /38,983	79,093 /6,172	534,525 /4,880
Completeness (%)	93.2 (92.4)	99.9 (99.6)	96.0 (86.8)
R_{sym} [b]	16.9 (66.5)	7.9 (56.1)	36.6 (124.2)
$CC_{1/2}$	0.97 (0.69)	0.99 (0.98)	0.90 (0.05)
Redundancy	2.0 (1.6)	12.8 (13.1)	12.2 (6.6)
I/sigma	15.6 (2.2)	25.8 (8.1)	18.9 (1.0)
**Refinement**			
R_{work}[c] / R_{free}[c] (%)	18.15/21.72	23.38/26.95	37.04/42.40
Protein atoms/DNA atoms	2,614	818	2,307/927
Water molecules	28	19	0
Average *B* value ($Å^2$)	25.54	33.97	288.16
r.m.s.d. bond (Å)	0.013	0.013	0.012
r.m.s.d. angle (°)	1.641	1.648	1.854
**Ramachandran plot (%)**			
Favoured	93.08	96.97	97.85
Allowed	1.60	3.03	1.08
Disallowed	0.32	0.00	1.08

Supplementary Table 2. Crystallographic data collection and refinement statistics.

[a] Numbers in parentheses indicate the statistics for the last resolution shell.

[b] $R_{sym} = \sum(|I_{hkl} - \langle I_{hkl} \rangle| / \sum \langle I_{hkl} \rangle$, where I_{hkl} = single value of measured intensity of *hkl* reflection, and $\langle I_{hkl} \rangle$ = mean of all measured value intensity of *hkl* reflection.

[c] $R_{work} = \sum(|F_{obs} - F_{calc}| / \sum F_{obs}$, where F_{obs} = observed structure factor amplitude, and F_{calc} = structure factor calculated from model. R_{free} is computed in the same manner as R_{work}, but from a test set containing 5% of data excluded from the refinement calculation.
